# Influence of reduced graphene oxide on the growth, structure and decomposition activity of white-rot fungus *Phanerochaete chrysosporium*

**DOI:** 10.1039/c7ra12364g

**Published:** 2018-01-29

**Authors:** Hua Yang, Shicheng Feng, Qiang Ma, Zhu Ming, Yitong Bai, Lingyun Chen, Sheng-Tao Yang

**Affiliations:** College of Chemistry and Environment Protection Engineering, Southwest Minzu University Chengdu 610041 China yangst@pku.edu.cn

## Abstract

Graphene materials have attracted great interest nowadays due to their large-scale production and wide applications. It is urgent to evaluate the ecological and environmental risk of graphene materials for the healthy development of the graphene industry. Herein, we evaluated the influence of reduced graphene oxide (RGO) on the growth, structure and decomposition activity of white-rot fungus, whose decomposition function is vital for carbon cycle. RGO slightly stimulated the fresh weight and dry weight gains of *Phanerochaete chrysosporium*. A larger number of fibrous structures were observed at low RGO concentrations in *P. chrysosporium*, which was consistent with the elongation of cells observed under a transmission electron microscope. RGO did not affect the chemical composition of *P. chrysosporium*. Moreover, the laccase production of *P. chrysosporium* was not influenced by RGO. The degradation activities of *P. chrysosporium* for dye and wood appeared to be promoted slightly, but the differences were insignificant compared to the control. Therefore, RGO had low toxicity to white-rot fungus and was relatively safe for the carbon cycle.

## Abstract 2

RGO stimulated the growth of white-rot fungus and did not influence its degradation activity.

## Graphical abstract

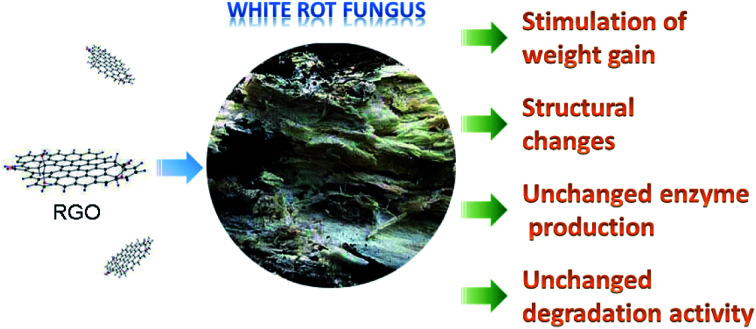

## Introduction

In recent years, graphene and its derivatives have attracted tremendous research interest due to their unique physical/chemical properties and potential applications in numerous fields.^1,2^ The intact graphene is formed solely by sp^2^ carbon atoms to produce six-membered rings.^3^ However, the chemical oxidation of graphite was the most convenient way to produce graphene oxide (GO) on a large scale, which could be further reduced to obtain reduced graphene oxide (RGO).^4^ Similar to intact graphene, RGO has a six-membered ring structure and excellent properties.^5^ The incomplete removal of oxygen usually leaves oxygen containing groups and defects on RGO. Currently, RGO has found applications in diverse areas, such as drug delivery,^6^ environmental remediation,^7^ energy conversion and charge storage,^8,9^ sensors^10,11^ and composite materials.^12,13^

With the increasing production and applications, the graphene-based materials would be released into the environment and cause potential hazards to the environment and human beings.^14,15^ The literature results have shown the potential environmental risks of graphene-based materials. For instance, early in 2011, we reported that GO induced oxidative stress to A549 cells in a dose-dependent manner.^16^ Zhao *et al.* reported that RGO was more toxic than GO to freshwater algae because the hydrophobic RGO more readily hetero-agglomerated with algae.^17^ RGO was reported to reduce the hatching rate of zebrafish embryos and the length of larvae, while carbon nanotubes (CNTs) and GO had limited impact.^18^ After oral exposure, RGO was reported to induce short-term decrease of locomotor activity and neuromuscular coordination, but did not affect anxiety-like, exploratory, or spatial learning and memory behaviours.^19^ In addition, the toxicity of GO and RGO might be associated with the binding of proteins; GO had much higher affinity and induced more structural changes to proteins.^20^

The carbon cycle is an important component of biogeochemical cycle.^21^ The decomposition is one of the key links in carbon cycle, due to which carbon returns into the atmosphere as CO_2_. Disruption of the decomposition would definitely affect the carbon cycle and cause serious hazards to the ecological environment.^22^ The main contributors of the decomposition are the microorganisms that decompose organic matter into CO_2_. For example, white-rot fungi could decompose lignin and cellulose; thus, they are the main decomposers of wood and straw.^23^ Recently, several groups reported that nanomaterials might disturb the enzyme production and decomposition activity of white-rot fungi. We have earlier reported that GO inhibited the growth of white-rot fungus *Phanerochaete chrysosporium* and led to the complete loss of decomposition activity at high GO concentrations.^24^ Zeng *et al.* reported that the toxicity of Ag nanoparticles (NPs) to *P. chrysosporium* was regulated by the sulfide source. The thioacetamide and NaHS promoted the activation of *P. chrysosporium* by citrate-Ag NPs at a higher concentration.^25^ Cysteine and Na_2_S induced different distribution and toxicity trends of Ag NPs in *P. chrysosporium* biofilm, where Na_2_S induced the aggregation and detoxification of Ag NPs.^26^ Ag NPs stimulated Cd(ii) removal by *P. chrysosporium* in aqueous solutions.^27^ In another study, Au NPs, CdSe/ZnS NPs, Mo/NaO NPs, and SDS/DDAB (sodium dodecyl sulfate/dimethyl dioctadecylammonium bromide) NPs significantly inhibited the growth of white-rot fungi with effects on the mycelium chemical composition.^28^ TiO_2_ NPs were found to prevent the growth of white-rot fungi in wood due to photocatalytic activity.^29^ Similarly, Ag NPs and Cu NPs improved the resistance of particleboard to *Trametes versicolor* fungus.^30^ When RGO enters the environment, it might also impact the function of white-rot fungi and disturb the carbon cycle just like other nanomaterials. However, such information is completely unknown to date, which hinders the environmental risk evaluation of graphene materials.

In this study, we investigated the influence of RGO on the growth, structure and decomposition activity of *P. chrysosporium*. The fresh weight and dry weight of *P. chrysosporium* were measured and the pH values of the culture system were recorded. The structural changes were investigated by observing the *P. chrysosporium* specimens under optical microscope, transmission electron microscope (TEM) and scanning electron microscope (SEM). The production of laccase (Lac) by *P. chrysosporium* was analysed. The decomposition performance of *P. chrysosporium* for dye and wood was evaluated. The implication to the environmental impact and safe applications of RGO are discussed.

## Materials and methods

### Preparation of RGO

GO was prepared by the modified Hummers method following our previous report.^7^ RGO was prepared by reducing GO *via* chemical reaction. Vitamin C was used as the reducing reagent because it is nontoxic and the reduction condition was mild.^31^ After gentle mixing, the vitamin C/GO mixture was incubated at 60 °C for 4 h at the mass ratio of 1 : 1 without stirring. A black RGO dispersion was formed without aggregation. The RGO dispersion was filtered with a filter paper (pore size: 0.45 mm) and the filter cake was washed with deionized water before lyophilisation. The obtained RGO was characterized by TEM (Tecnai G2 20, FEI, USA), X-ray photoelectron spectroscopy (XPS, Kratos, UK), infrared spectroscopy (IR, Magna-IR 750, Nicolet, USA) and Raman spectroscopy (inVia, Renishaw, UK).

### Culture of *P. chrysosporium*

The fungal strain *P. chrysosporium* (MTCC 787) was obtained from the Guangdong Microbiology Culture Centre, Guangzhou, China. The fungus was grown on glucose potato agar plates at 37 °C for 7 d. For inoculum, the fungal spore dispersion was prepared by harvesting all the fungal spores from one 7 d-old agar plate and dispersing them into 10 mL of liquid medium. Fungi were grown in Erlenmeyer flasks with 40 mL of medium for each. The flasks were inoculated at the density of 5 × 10^5^ spore per mL and incubated at 37 °C on a rotating orbital incubator shaker at 100 rpm.

The liquid medium was composed of the following: glucose (10 g L^−1^), KH_2_PO_4_ (2.56 g L^−1^), MgSO_4_·7H_2_O (0.71 g L^−1^), ammonium tartrate (0.2 g L^−1^), benzyl alcohol (0.54 g L^−1^), thiamine (0.001 g L^−1^), trace element solution (70 mL L^−1^), acetic acid (0.9 g L^−1^) and sodium acetate (0.9 g L^−1^). After adjusting the pH to 5.0 with acetic acid, the medium was sterilized before use. The components of trace element solution were: glycine (0.6 g L^−1^), MnSO_4_·H_2_O (0.5 g L^−1^), NaCl (1 g L^−1^), FeSO_4_·7H_2_O (0.1 g L^−1^), CoCl_2_·H_2_O (0.19 g L^−1^), CaCl_2_·2H_2_O (1.56 g L^−1^), ZnSO_4_·7H_2_O (0.1 g L^−1^), CuSO_4_·5H_2_O (0.1 g L^−1^), KAl(SO_4_)_2_·12H_2_O (0.01 g L^−1^), HBO_3_ (0.01 g L^−1^), and Na_2_MoO_4_·2H_2_O (0.01 g L^−1^).^24^

### Influence of RGO on the growth and structure of *P. chrysosporium*

To investigate the influence on the fungus growth, the culture medium was supplemented with RGO at the concentrations of 0, 0.25, 0.5, 0.75, 1.0, 2.0, 3.0, and 4.0 mg mL^−1^. The concentration range was selected to compare the results with our previous report (0–4 mg mL^−1^) and it already covered the most frequently studied concentrations in the literature.^24,32^ The pH values of the culture media were adjusted to 5.0 and measured using a pH meter (PB10, Sartorius Co., Germany). The spores were inoculated at the density of 5 × 10^5^ spores per mL. The flasks were incubated at 37 °C on a rotating orbital incubator shaker at 100 rpm. After 14 d incubation, the *P. chrysosporium* were filtered using a filter paper (pore size of 0.45 mm). The pH values of the culture media were measured again. The fresh weight of *P. chrysosporium* was recorded by removing the water with filter paper. The dry weight was measured after drying in vacuum oven at 60 °C for 48 h to ensure the complete removal of water. Herein, 14 d-observation period was selected because *P. chrysosporium* produces lots of enzymes at 14 d.

Another set of exposure was performed as aforementioned and the fresh *P. chrysosporium* samples were collected for structural observations. For optical microscopy, the samples were fixed with 4% formaldehyde, embedded in paraffin, thin-sectioned, mounted on glass microscope slides, and then stained with periodic acid Schiff (PAS) stain method.^24^ The photographs were recorded under a microscope equipped with a charge coupled device (CAB-30PC, Carbontek Co., Chengdu, China). For TEM observations, the samples were fixed with 2.5% glutaraldehyde, post-fixed in 1% osmium tetroxide, dehydrated in a graded alcohol series, embedded in epoxy resin, and cut with an ultramicrotome. Thin sections post-stained with uranyl acetate and lead citrate were observed under TEM (Tecnai G2 20, FEI, USA).^16^ For SEM observations, the fresh *P. chrysosporium* samples were lyophilised and coated with gold for 5 s using a sputter coater (JFC 1600, JEOL, Japan). The samples were examined using a scanning electron microscope (SEM, S-4800, Hitachi, Japan).^33^

### Effect of RGO on Lac activity

The culture medium was supplemented with RGO (0–4 mg mL^−1^) and *P. chrysosporium* was incubated at 37 °C on a rotating orbital incubator shaker at 100 rpm. After 14 d-incubation, the fermentation broth was centrifuged at 4000 rpm for 10 min to collect the supernatant for the Lac activity assay. Determination of Lac activity was performed with 2,2′-azino-bis(3-ethylbenzothiazoline-6-sulfonic) (ABTS, bought from Beijing Solarbio Science & Technology Co., Ltd., China) following the literature method.^34^

### Influence of RGO on the decomposition activity

For dye decomposition, the spores were inoculated at the density of 5 × 10^5^ spore per mL in the RGO containing media (0–4 mg mL^−1^). The flasks were incubated at 37 °C on a rotating orbital incubator shaker at 100 rpm. At day 3, reactive brilliant red X-3B (Shanghai Citailong Co. Ltd., China) was introduced to the flasks at the final concentration of 30 mg L^−1^. The system was incubated for another 4 d and the absorbance of the supernatant was recorded at 538 nm on a UV-vis spectrometer (UV1600, Shanghai Mapada Instruments Co., China) for the calculation of decoloration efficiency.^24^

For the decomposition of wood, the culture medium (12 mL) was inoculated with 1 × 10^7^ spores, supplemented with sawdust (5 g, bought from Dashuo Experimental Animal Co., Chengdu, China) and different amounts of RGO (0, 0.01, 0.02, 0.04, 0.16 g) and then placed in a dark/static incubator at 37 °C for static fermentation.^29^ The liquid medium was composed of the following: yeast (5 g L^−1^), KH_2_PO_4_ (2 g L^−1^), and MgCl_2_ (1 g L^−1^). After 90 d-incubation, the dry weight of sawdust was recorded after drying in vacuum oven at 60 °C for 48 h. The samples were examined under SEM to demonstrate the breakage of wood surface.

### Statistical analysis

All data were expressed as the mean of individual observations with standard deviation (mean ± SD). Significance was calculated using Student's *t*-test, where *p* < 0.05 was considered as the statistical significance (indicated as *).

## Results and discussion

### Characterization of RGO

**Fig. 1:**
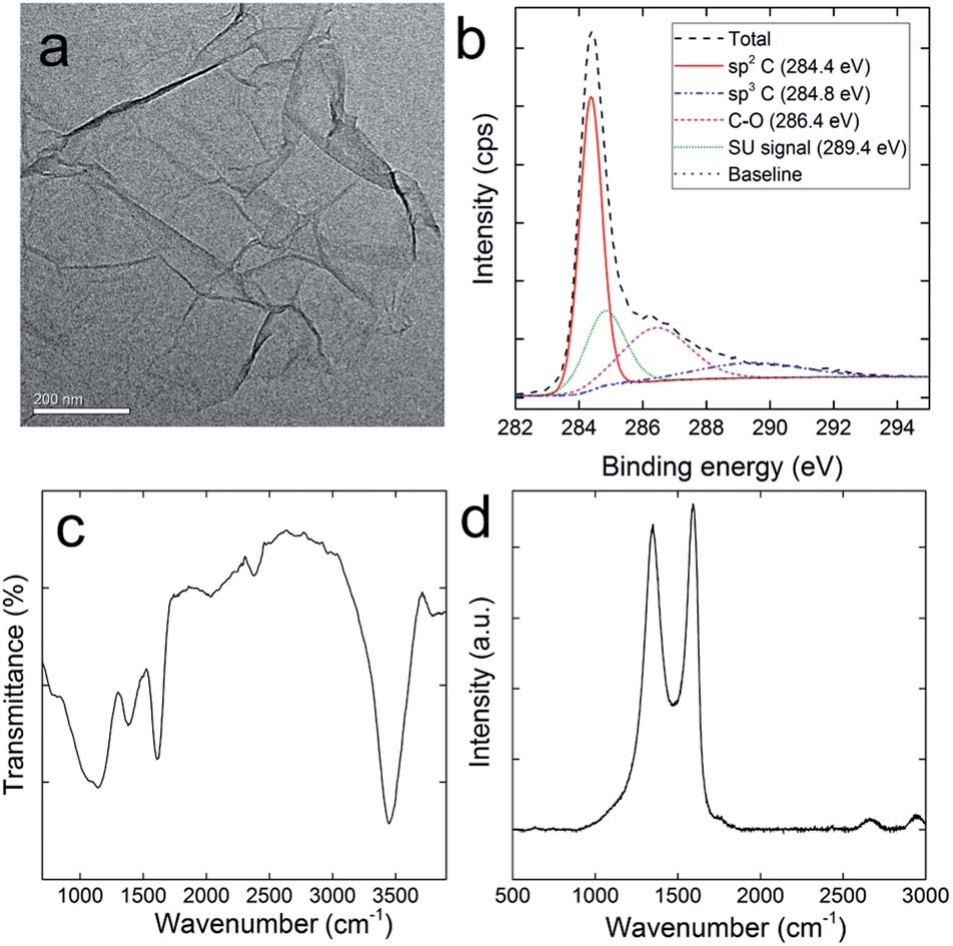
Characterization of RGO: (a) representative TEM image; (b) C 1s XPS spectrum; (c) IR spectrum; (d) Raman spectrum.

RGO was carefully characterized before use to ensure the purity and functionalities. As shown in Fig. 1a, the typical sheet structure of graphene was observed for RGO. The sheet stretched effectively with slight folding. The chemical components of RGO were analysed *via* XPS. There were 83.6 at% of carbon atoms, 15.1 at% of oxygen atoms, and 1.3 at% of nitrogen atoms in RGO. The carbon atoms could be divided into three components (Fig. 1b), namely, sp^2^ C (50.6%), sp^3^ C (22.6%) and C–O (26.8%). The shake-up (SU) signal was observed at 290.4 eV, which was also observed in other carbon nanomaterials.^35^ Comparing with the data of GO, the reduction by ascorbic acid was efficient and consistent with the literature results.^36^ The remaining oxygen atoms were in the forms of oxygen containing groups, which could be distinguished in the IR spectrum (Fig. 1c). The broad band at around 3450 cm^−1^ was assigned to the –OH groups. The narrow band at 1610 cm^−1^ was assigned to the C=C skeletal stretching vibrations. No signal was detected at around 1720 cm^−1^, suggesting the absence of C=O, which was consistent with the C 1s XPS result. In addition, the typical absorption bands of ascorbic acid could not be observed in the IR spectrum, indicating the complete removal of ascorbic acid after washing. The typical Raman spectrum of RGO was recorded to confirm the sp^2^ carbon rings and defects (Fig. 1d). The D band (1346 cm^−1^) and G band (1593 cm^−1^) were clearly recognized. The D band indicated the structural defects and disorder, whereas the G band represented the graphitic structure. The intensity ratio of the D band and G band (*I*_D_/*I*_G_) of RGO is 0.81, suggesting that the disorder of RGO structure was mild. Overall, the characterization results collectively suggested that RGO sample was effectively reduced with no heavy metal or ascorbic acid impurity; thus, it was suitable for the toxicity evaluations.

### Growth of *P. chrysosporium*

**Fig. 2:**
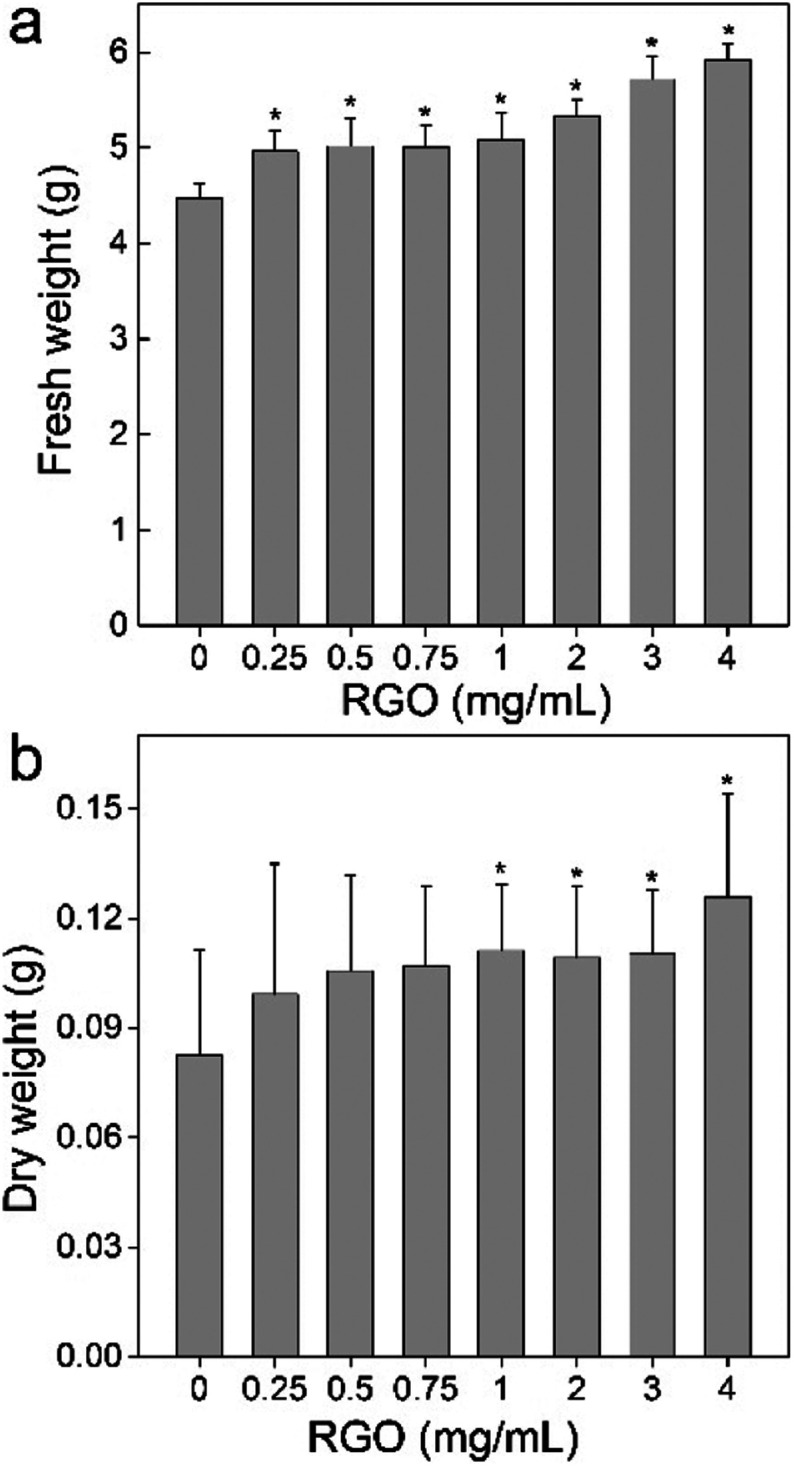
Influence of RGO on the weight gain of *P. chrysosporium* (*n* = 4): (a) fresh weight; (b) dry weight.

RGO showed the stimulating effect to *P. chrysosporium* according to the fresh- and dry-weight increase. The fresh weights of *P. chrysosporium* were significantly higher than that of the control group after the 14 d-incubation at all RGO concentrations (Fig. 2a). The enhancement of fresh weight was dose-dependent. The fresh weight was 111% of the control at RGO concentration of 0.25 mg mL^−1^ and further increased to 132% at 4 mg mL^−1^. It should be noted that RGO was non-dispersible in aqueous systems, where part of RGO was wrapped by the fungi and the rest precipitated. It was not possible to determine the actual exposure concentrations, so the concentrations were expressed by dividing the weight of added RGO with medium volume. For dry weight, the situation was similar, but the enhancement became significant only at RGO concentration of 1 mg mL^−1^ or higher (Fig. 2b). The fresh weight was 120% of the control at RGO concentration of 0.25 mg mL^−1^ and further increased to 152% at 4 mg mL^−1^. Based on the fresh and dry weights, RGO had higher influence on the dry weight, corresponding to the decrease of water content of *P. chrysosporium*. The influence of RGO on *P. chrysosporium* growth was different to that of GO. Previously, we reported that GO stimulated the growth (123% of the control) of *P. chrysosporium* at low concentrations (0.1 mg mL^−1^) and severely inhibited the weight gain at high concentrations (2–4 mg mL^−1^).^24^ Although RGO appeared to have higher nontoxic concentrations in this study, it should be noticed that RGO was non-dispersible in the medium and the aggregation of RGO would reduce the practical exposure concentrations. In addition, RGO had lower binding affinity to proteins and less impact on the protein structures;^20^ therefore, the lower toxicity of RGO was not surprising.

**Fig. 3:**
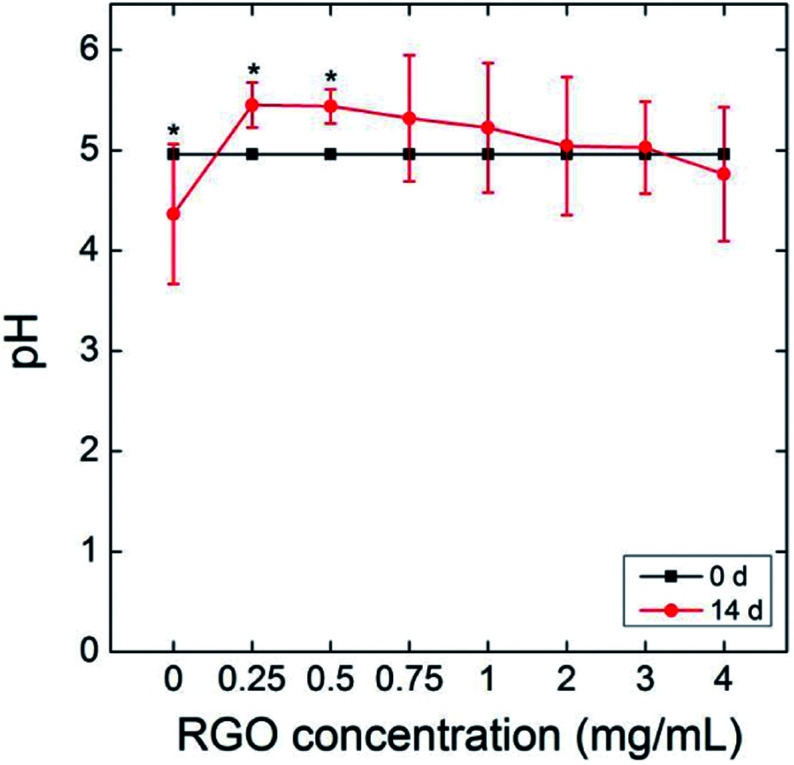
Influence of RGO on the pH value of culture system (*n* = 4).

A possible explanation could be that RGO does not possess abundant carboxyl groups, which could deprotonate to release H^+^. The pH value of the culture system decreased from 5.0 to 4.4 (Fig. 3), where the acidification was due to the metabolism of *P. chrysosporium*. With the addition of RGO, the pH values showed a statistically significant increase at 0.25 and 0.5 mg mL^−1^ ( *p* < 0.05). At even higher RGO concentrations, the pH values were similar to the starting pH value ( *p* > 0.05). Moreover, GO induced the pH decrease of 1 at 4 mg mL^−1^, so more acidic substances might inhibit the *P. chrysosporium* growth.^24^

### Structural changes

**Fig. 4:**
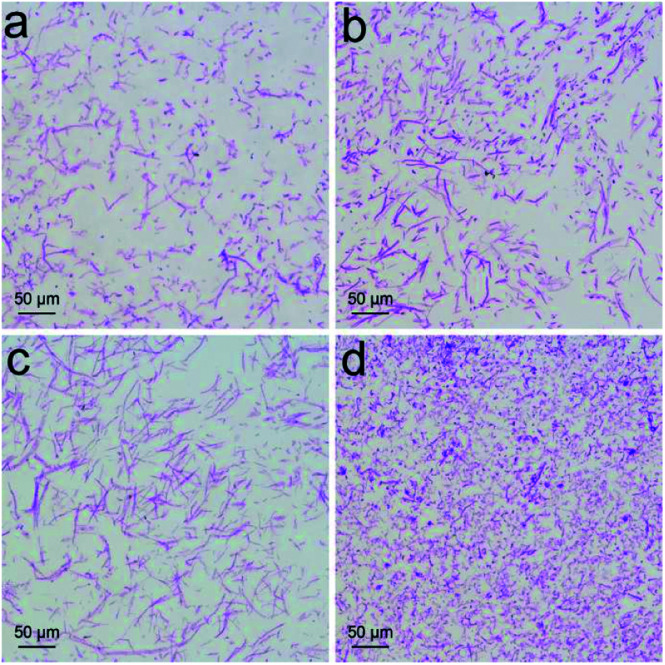
PAS staining of *P. chrysosporium* in the absence (a) and presence of 0.25 mg mL^−1^ (b), 1.0 mg mL^−1^ (c) and 4.0 mg mL^−1^ (d) of RGO.

Increased weight gain of *P. chrysosporium* generally indicated the nontoxic nature of RGO. More precise investigation was performed focusing on the structural changes that were more sensitive to the toxicant. First, we checked the fibrous structure of *P. chrysosporium* under optical microscope (Fig. 4). The macro-morphology was not influenced except the colour turned grey, which was due to the absorbance of RGO. Upon PAS staining, glycogen was oxidized by periodic acid and stained by Schiff reagent to present fuchsia colour. As shown in Fig. 4a, thin fibres were observed in the control group with the fibre lengths of 20–50 μm (31 ± 16 μm), obtained on measuring 50 fibre lengths. With the addition of RGO (0.25 mg mL^−1^), the fibre lengths increased (32 ± 18 μm) and the amount of amorphous structures decreased (Fig. 4b). The lengths of the fibres further increased to around 60 ± 15 μm at RGO concentration of 1.0 mg mL^−1^, but the fibres looked thicker at that concentration (Fig. 4c). The majority of *P. chrysosporium* became amorphous (Fig. 4d) when the RGO concentration reached 4.0 mg mL^−1^. The fibre lengths decreased to around 17 ± 9 μm. Therefore, although RGO showed a stimulating effect on the weight gain, it altered the fibrous structure of *P. chrysosporium*. Again, this phenomenon was different to that exposed to GO.^24^ GO disturbed the fibre formation and some very long fibres formed at high concentrations (2.0 and 4.0 mg mL^−1^), while most of the *P. chrysosporium* became amorphous.

**Fig. 5:**
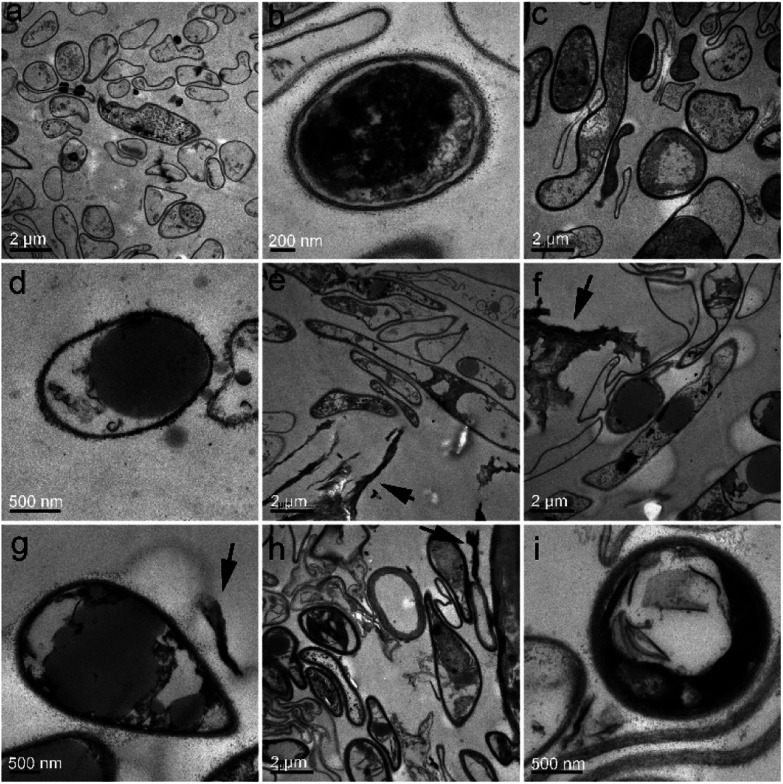
TEM images of *P. chrysosporium* in the absence (a and b) and presence of 0.25 mg mL^−1^ (c and d), 1.0 mg mL^−1^ (e–g) and 4.0 mg mL^−1^ (h and i) of RGO. The extracellular RGO is indicated by black arrows.

For closer observations, we used TEM to investigate the ultrastructural changes of *P. chrysosporium*. Small oval-shaped cells were observed in the control group and the diameters of the cells were about 1.5 μm (Fig. 5a). The cell wall and membrane were easily distinguished as intact and close (Fig. 5b). Even at the low RGO concentration (0.25 mg mL^−1^), the ultrastructure of *P. chrysosporium* changed significantly. As shown in Fig. 5c, the shape of the cells became irregular and there were very large/small cells observed. The cell membrane and wall became fuzzy (Fig. 5d). At RGO concentration of 1.0 mg mL^−1^, *P. chrysosporium* cells showed higher aspect ratios (Fig. 5e–g). The longest ones were even about 10 μm in length. The extracellular RGO could be distinguished, but none was observed intracellular, suggesting that RGO hardly entered the *P. chrysosporium* cells. At high RGO concentration of 4.0 mg mL^−1^, the *P. chrysosporium* cells remained irregular, but the aspect ratio decreased (Fig. 5h and i). This was consistent with the optical observations that fewer fibres formed at 4.0 mg mL^−1^.

**Fig. 6:**
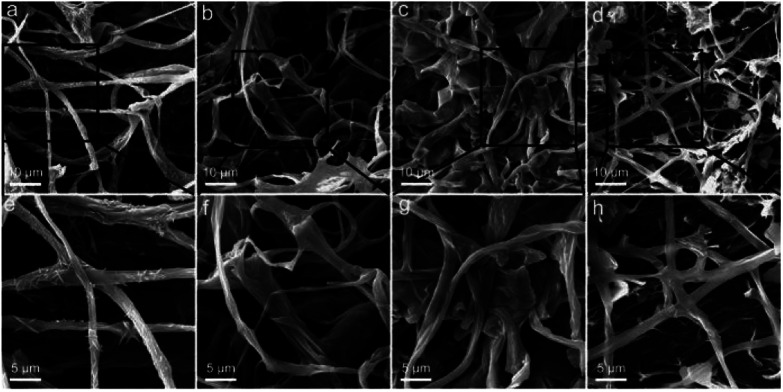
SEM images of *P. chrysosporium* in the absence (a and e) and presence of 0.25 mg mL^−1^ (b and f), 1.0 mg mL^−1^ (c and g) and 4.0 mg mL^−1^ (d and h) of RGO.

PAS staining and TEM observation required the slicing of samples and only reflected the inner structures; thus, the surface morphology was further investigated by SEM. The typical fibrous structures were clearly recognized in the control group (Fig. 6a and e). The acicular particles were observed around the mycelium, which were assigned to the inorganic salts in the culture medium. The mycelium was smooth and cross-linked. At RGO concentration of 0.25 mg mL^−1^, the fibres became thicker and broadened (Fig. 6b and f). The inorganic salts seemed to disappear on the mycelium surface. Instead, some flat graphene sheets bound on the mycelium surface. At RGO concentration of 1.0 mg mL^−1^, similar phenomena were observed with more RGO bound to the mycelium (Fig. 6c and g). However, further increase of RGO concentration to 4.0 mg mL^−1^ led to the decrease of fibre density and the widths (Fig. 6d). The mycelium was thinner as compared to the other groups (Fig. 6h). Again, SEM observations supported the conclusion that RGO stimulated the formation of fibres at low concentrations and inhibited the same at high concentration.

### Decomposition activity

**Fig. 7:**
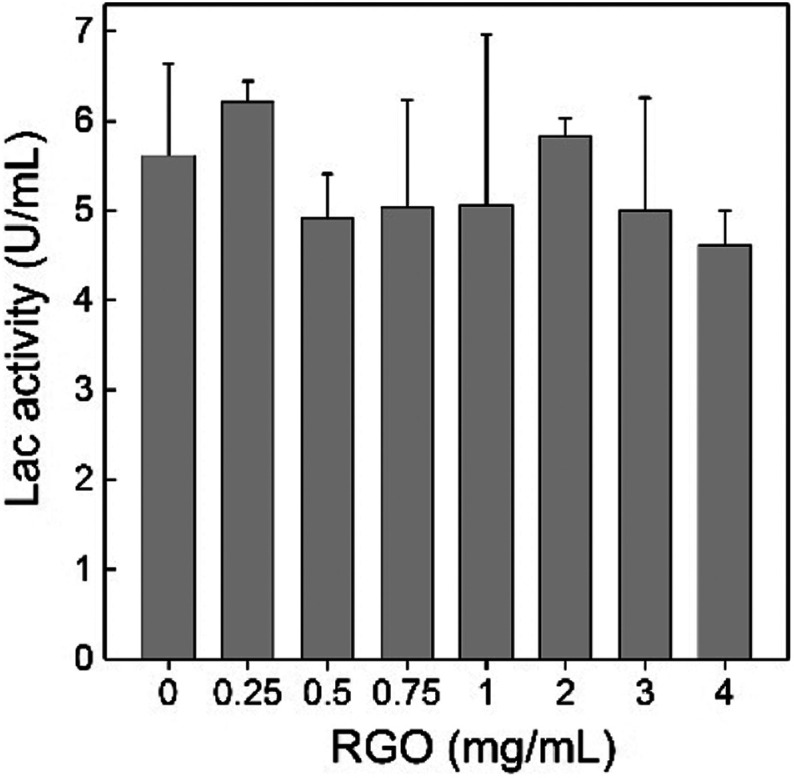
Effect of RGO on the Lac production of *P. chrysosporium* (*n* = 3).

*P. chrysosporium* decomposes lignin primarily through the secretions of Lac, manganese peroxidase and ligninase. The exposure to RGO might affect the decomposition activity of *P. chrysosporium* by inhibiting the enzyme activity. Herein, we analysed the Lac activity of the culture system after the exposure to RGO (Fig. 7). At all RGO concentrations, Lac activities of *P. chrysosporium* were not affected by RGO ( *p* > 0.05). Considering that RGO had negligible influence of enzyme activity in solution,^24^ the unchanged Lac activity implied that the secretion of Lac was not affected by RGO. This was consistent with the literature results. Rodriguez-Couto used RGO hydrogel and xerogel to support the growth of white rot fungus *Trametes pubescens*.^37^ RGO hydrogel did not affect the Lac activity of *T. pubescens*, while RGO xerogel showed significant stimulating effect. In addition, the functional degree of carbon nanomaterials should be considered as the regulating parameter of enzyme production of white-rot fungi. Berry *et al.* reported that pristine CNTs did not change the enzyme activity of *T. versicolor* and *Phlebia tremellosa*, while carboxylated CNTs enhanced the enzyme production.^38^

**Fig. 8:**
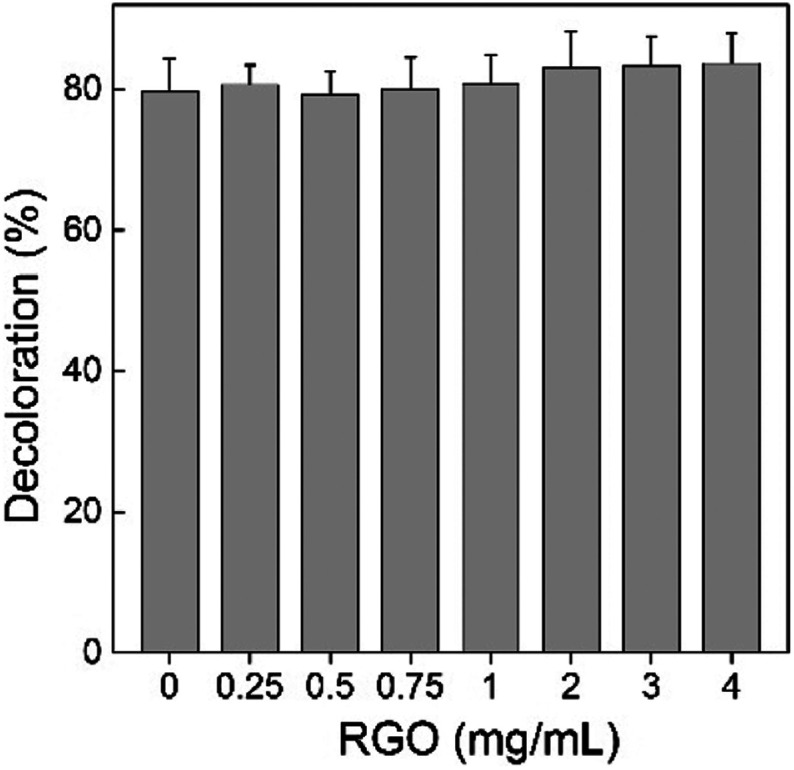
Influence of RGO on the decomposition activity of *P. chrysosporium* for reactive brilliant red X-3B (*n* = 3).

First, we tested the decomposition activity of *P. chrysosporium* by monitoring the decoloration of reactive brilliant red X-3B. As shown in Fig. 8, the decoloration capability of *P. chrysosporium* was not affected by RGO in the test concentration range ( *p* > 0.05). Reactive brilliant red X-3B was the typical indicator for the decomposition activity of *P. chrysosporium*; therefore, the results suggested that RGO did not change the activity of *P. chrysosporium*. This was quite different to that of GO, where GO led to the complete loss of activity at concentrations of 1.0 mg mL^−1^ and higher.^24^ In another study, it was observed that Fe_2_O_3_ NPs slightly increased the degradation of bisphenol A by *Pleurotus ostreatus*.^39^

**Fig. 9:**
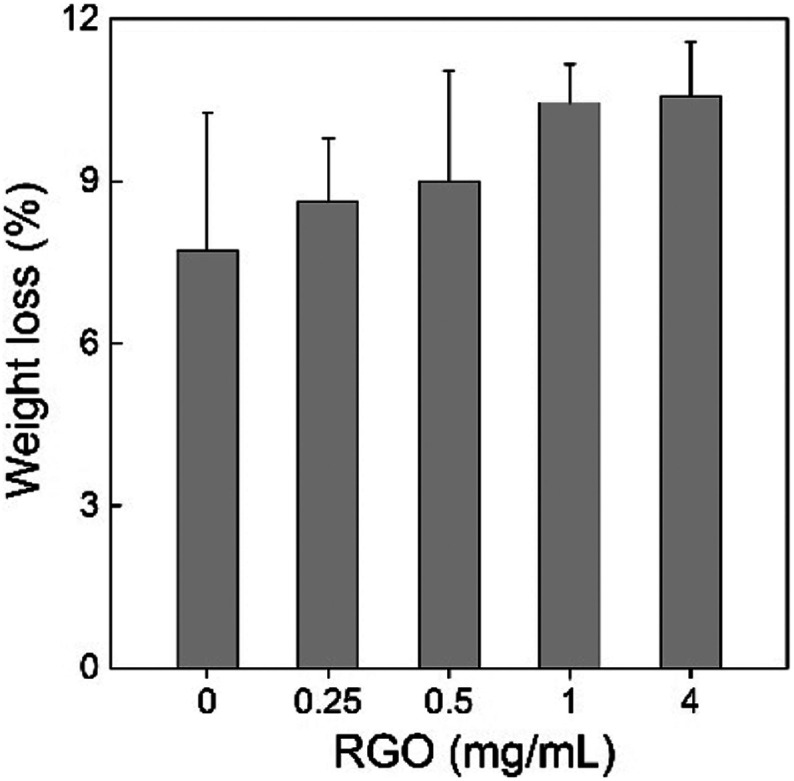
Weight loss of the sawdust after the degradation by *P. chrysosporium* in the absence/presence of RGO (*n* = 4).

Second, we measured the weight loss of sawdust in the *P. chrysosporium* culture systems in the presence/absence of RGO. The degradation of wood and straw is the most important ecological function of *P. chrysosporium* as the decomposer. The alteration of the degradation activity would disturb the ecological balance and the carbon cycle. According to the weight loss rate, RGO induced no statistically significant change to the degradation activity of *P. chrysosporium* for sawdust ( *p* > 0.05) although a slight increase trend was observed (Fig. 9). More weight loss was observed at higher RGO concentration. Based on IR analyses, no visible change in the chemical compositions of sawdust was observed (data not shown). In the literature, TiO_2_ NPs, Cu NPs and Ag NPs were found to inhibit wood degradation by white-rot fungi.^29,30,40^ Thus, the influence of nanomaterials on the degradation of wood depended on the properties of nanomaterials. In this regard, RGO was more environment-friendly than the aforementioned metal containing NPs.

**Fig. 10:**
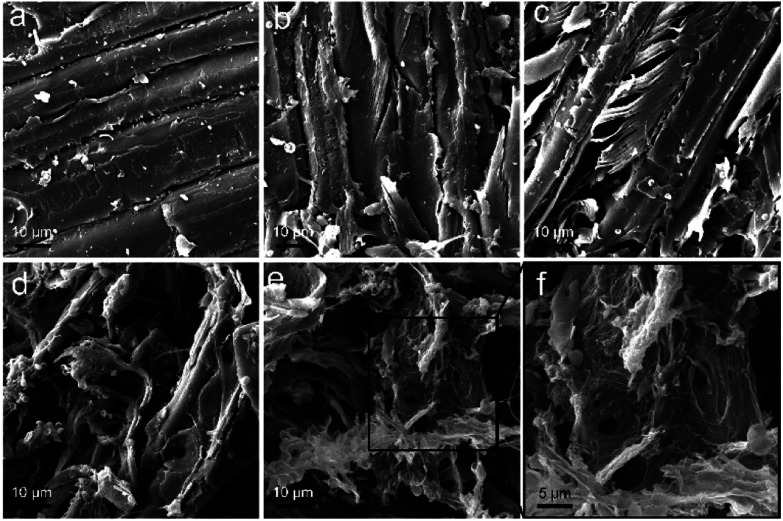
SEM images of the sawdust before (a), and after the degradation by *P. chrysosporium* in the absence (b) and presence of 0.25 mg mL^−1^ (c), 1.0 mg mL^−1^ (d) and 4.0 mg mL^−1^ (e and f) of RGO.

The degradation of sawdust by *P. chrysosporium* was directly indicated by SEM observations. The intact sawdust before degradation is shown in Fig. 10a. When sawdust was incubated with *P. chrysosporium*, the fibre cell walls were partially degraded into smaller pieces and some cracks were formed (Fig. 10b). With the addition of RGO (0.25 mg mL^−1^), the degraded area of sawdust expanded (Fig. 10c). More *P. chrysosporium* spores were recognized as the bowl-shaped spots. It seemed that RGO increased the attachment of *P. chrysosporium* on sawdust. The phenomenon was more evident at RGO concentration of 1.0 and 4.0 mg mL^−1^ (Fig. 10d–f). In particular, as shown in Fig. 10f, the middle lamella was crowded with *P. chrysosporium* fibres and spores. Thus, although RGO did not enhance the wood degradation, RGO led to the tighter binding of *P. chrysosporium* to the sawdust surface. In contrast, when the wood was incubated with TiO_2_ NPs, the lower degradation was reflected by the more intact wood surface under SEM.^29^

### Implications

White-rot fungi are the main organisms that decompose lignin, which is crucial in maintaining the carbon cycle.^41^ When RGO enters the environment, it might interact with white-rot fungi. Our results suggested that RGO enhanced the growth of white-rot fungi, disturbed the structures, but did not change the degradation activity.

Mechanistically, the toxicity of RGO to white-rot fungi might be due to three contributors. First, oxidative damage was the widely reported toxicological mechanism of nanomaterials.^16,42,43^ The literature also confirmed that Ag NPs induced oxidative stress to white-rot fungi.^25,26^ Second, the high strength of RGO might induce physical damage to *P. chrysosporium*, similar to that observed in case of CNTs; CNTs have been reported to penetrate the bacterial membrane as a dart.^44^ Third, RGO had high absorption capacity, which might lead to the depletion of nutrition components. This pathway was observed for GO in cellular evaluations.^45^ Further investigations are required to clarify the toxicological mechanism in future.

On comparing the results of GO and RGO,^24^ we could clearly see the importance of chemical reduction on graphene toxicity to white-rot fungi. As aforementioned, the chemical reduction had several influences on the graphene properties.^7,20^ First, the chemical reduction led to the precipitation of RGO. This definitely reduced the direct contact of RGO with white-rot fungi. The lower exposure would surely alleviate the environmental risk of RGO. Second, the chemical reduction removed the carboxyl groups, which might release H^+^ due to the deprotonation of carboxyl groups. The acidification of culture system by GO might be one of the toxicological pathways. In contrast, no carboxyl group was detected in RGO; thus, the acidification did not occur. Third, RGO had limited interaction with proteins according to enzyme activity measurements, circular dichroism spectra and fluorescence spectra;^20^ thus, it had less impact on protein structure and functions. Based on these observations, the lower toxicity of RGO was reasonable. When applying graphene-based materials, the reduction degree should be considered as the approach to control the environmental hazards.

Since white-rot fungi are the decomposers in carbon cycle, the degradation activity of white-rot fungi is crucial in evaluating the nanoimpact of graphene materials. The available results in literature indicated that different nanomaterials had different impact on the degradation activity of white-rot fungi.^24–30^ In addition, external substances, such as metal impurities and sulfide, also had significant influence on the enzyme activity of white-rot fungi.^25–27^ Furthermore, due to the lower toxicity and protein affinity, RGO had no influence on the degradation function of white-rot fungi. When RGO was released into the environment, it would most probably have no influence the function of white-rot fungi. Therefore, RGO might not disturb the decomposition of carbon cycle. In the future, the co-exposure of RGO and other toxic/nontoxic substances should be evaluated since the environmentally exposed pollutants usually contain multiple substances. In addition, the degradation of graphene by enzymes from white-rot fungi was reported.^46^ The biotransformation or degradation of graphene in white rot fungi culture systems should be further evaluated.

## Conclusions

In summary, RGO showed low toxicity to white-rot fungus throughout the evaluations on weight gain, structure, enzyme production, and degradation activity. RGO stimulated the growth of *P. chrysosporium* slightly according to the fresh-weight and dry-weight evaluations. RGO promoted the fibrous structure formation at low concentrations and the elongated cells under TEM supported this finding. The production of Lac was not influenced by RGO, which consequently led to the unchanged degradation activity of *P. chrysosporium* for dye and wood. Therefore, from the perspective of decomposition (one crucial link of carbon cycle), RGO had no significant influence on the decomposition activity of white-rot fungus and thus, it was of low risk to the carbon cycle. We hope that our obtained results would benefit the environmental risk evaluations of graphene materials and stimulate more efforts in the nanoimpact on the biogeochemical cycle.

## Conflicts of interest

The authors report no conflicts of interest in this work.

## Supplementary Material
